# Nurse-Moderated Internet-Based Support for New Mothers: Non-Inferiority, Randomized Controlled Trial

**DOI:** 10.2196/jmir.6839

**Published:** 2017-07-24

**Authors:** Michael G Sawyer, Christy E Reece, Kerrie Bowering, Debra Jeffs, Alyssa C P Sawyer, Murthy Mittinty, John W Lynch

**Affiliations:** ^1^ School of Medicine University of Adelaide Adelaide Australia; ^2^ Research and Evaluation Unit Women's and Children's Health Network Adelaide Australia; ^3^ Child and Family Health Service Women's and Children's Health Network Adelaide Australia; ^4^ SA State Office Department of Social Services Adelaide Australia; ^5^ School of Public Health University of Adelaide Adelaide Australia; ^6^ School of Social and Community Medicine University of Bristol Bristol United Kingdom

**Keywords:** public health informatics, community health services, Internet

## Abstract

**Background:**

Internet-based interventions moderated by community nurses have the potential to improve support offered to new mothers, many of whom now make extensive use of the Internet to obtain information about infant care. However, evidence from population-based randomized controlled trials is lacking.

**Objective:**

The aim of this study was to test the non-inferiority of outcomes for mothers and infants who received a clinic-based postnatal health check plus nurse-moderated, Internet-based group support when infants were aged 1-7 months as compared with outcomes for those who received standard care consisting of postnatal home-based support provided by a community nurse.

**Methods:**

The design of the study was a pragmatic, preference, non-inferiority randomized control trial. Participants were recruited from mothers contacted for their postnatal health check, which is offered to all mothers in South Australia. Mothers were assigned either (1) on the basis of their preference to clinic+Internet or home-based support groups (n=328), or (2) randomly assigned to clinic+Internet or home-based groups if they declared no strong preference (n=491). The overall response rate was 44.8% (819/1827). The primary outcome was parenting self-competence, as measured by the Parenting Stress Index (PSI) Competence subscale, and the Karitane Parenting Confidence Scale scores. Secondary outcome measures included PSI Isolation, Interpersonal Support Evaluation List–Short Form, Maternal Support Scale, Ages and Stages Questionnaire–Social-Emotional and MacArthur Communicative Development Inventory (MCDI) scores. Assessments were completed offline via self-assessment questionnaires at enrolment (mean child age=4.1 weeks, SD 1.3) and again when infants were aged 9, 15, and 21 months.

**Results:**

Generalized estimating equations adjusting for post-randomization baseline imbalances showed that differences in outcomes between mothers in the clinic+Internet and home-based support groups did not exceed the pre-specified margin of inferiority (0.25 of a SD) on any outcome measure at any follow-up assessment, with the exception of MCDI scores assessing children’s language development at 21 months for randomized mothers, and PSI Isolation scores at 9 months for preference mothers.

**Conclusion:**

Maternal and child outcomes from a clinic-based postnatal health check plus nurse-moderated Internet-based support were not inferior to those achieved by a universal home-based postnatal support program. Postnatal maternal and infant support using the Internet is a promising alternative to home-based universal support programs.

**Trial Registration:**

Australian New Zealand Clinical Trials Registry Number (ANZCTR): ACTRN12613000204741; https://www.anzctr.org.au/Trial/Registration/TrialReview.aspx?id=363712&isReview=true (Archived by WebCite at http://www.webcitation.org/6rZeCJ3k1)

## Introduction

Universal home-based support for mothers and infants has been a core component of health system outreach since the mid-late 19th century in the United Kingdom, and since the 1930s in European welfare states such as Denmark, Finland, and the Netherlands [1-3]. Postnatal home-based support designed to engage the entire newborn population is used to screen for the presence of maternal and infant problems and to offer support using principles of proportionate universal service delivery—more support for those with greater need [4]. In contemporary Australia, as well as checking maternal and infant health, nurses providing home-based support promote parent knowledge and positive attitudes relevant to child rearing and refer mothers and infants who require additional help to appropriate specialist services, including more intensive nurse home-visiting programs [5,6]. There are clearly advantages to home-based nurse support. It allows nurses to observe the conditions in the home and surrounding area and may feel like a more convenient, natural, and comfortable method of support for mothers. On the other hand, home visits are an expensive way to deliver nurse-led programs to whole populations and involve substantial transport and travel time costs, especially in more geographically dispersed urban and rural environments.

In the past, home-based support provided by community nurses was a key source of information and professional support for mothers of young children. However, the Internet now provides a convenient and private source of health information, the opportunity to exchange information with other mothers, and access to interactive treatment programs designed to address problems such as depression or anxiety [7-10]. Use of the Internet among women of child-bearing age in Australia and many other countries is now ubiquitous [7,11]. This offers health systems new opportunities to better support mothers in an equitable and potentially cost effective manner.

Increasing use of the Internet by mothers to obtain information and support has encouraged the development of numerous Websites and “mobile phone apps” by non-government and commercial organizations [12,13]. However, a concern for professionals and mothers is the variable quality of information provided on Internet websites and their lack of connection with local health services [9,13,14]. For example, it has been reported that health-related information on the Internet can be misleading and occasionally, “utterly wrong” [9,15,16]. In contrast, offering information within the context of professional evidence-based nurse support to mothers via the Internet has the potential to help address concerns about the quality of information and advice provided. Furthermore, if support is offered in a nurse-led group-based format it provides mothers with access to both professional and peer support during the immediate postnatal period. We know of no other interventions that use the Internet as a source of information and social support in combination with professional nurse support.

A recent systematic review of studies between 1998 and 2012 that compared outcomes for postpartum home-based with clinic-based support (without an Internet component) for mothers and infants drew attention to the paucity of studies and inconsistency of results in this area [17]. Conclusions of this review suggested that mothers preferred traditional home-based support to clinic-based care, and those receiving home-based support may persist longer with breastfeeding. Other studies have reported that mothers receiving home-based support have fewer acute care visits, re-hospitalizations and missed well-baby visits than those receiving either no follow-up or telephone follow-up post-delivery [18,19]. However, information in the area is very limited and no previous studies to our knowledge have compared home-based support to clinic-based care combined with Internet-based support provided by community nurses.

In close collaboration with the South Australian Child and Family Health Service (CaFHS), we devised a clinic+Internet intervention that would be achievable by nurses, feasible within current service delivery, and consistent with clinical governance models. As this is the first study of its kind, we utilized a non-inferiority design because of the strong clinical, social, and political support for home-based support programs, and concern that a clinic-based postnatal health check plus Internet-based support may generate inferior outcomes to that achieved by home-based support [20]. Thus, the first research task was to test whether similar outcomes could be achieved with clinic+Internet support versus traditional home-based support. We additionally included a preference trial arm to examine whether there were differences among those who would choose either the home-based or clinic+Internet service. This preference trial arm is essentially an observational study run in parallel with the randomized controlled trial (RCT). The design facilitates greater external validity of results than those based only on participants willing to be randomized, who may differ from those who have a strong preference for one or other of the interventions offered during the consent process.

This study compares maternal and infant outcomes for those who received the new Internet-based intervention versus outcomes for those who received standard postnatal home-based support from a community nurse, routinely offered to all mothers in South Australia (Australian New Zealand Clinical Trials Registry ACTRN12613000204741). We have previously reported intervention mothers’ level of engagement with the website [21].

## Methods

### Participants, Recruitment, and Randomization

Participants were new mothers referred by their birthing hospital to 1 of 6 CaFHS community clinics in Adelaide, South Australia, for their initial postnatal health check. During March to December, 2013, when CaFHS administrative officers from these clinics telephoned mothers to arrange their health check, they informed mothers about the study and sought verbal consent for the research team to contact mothers. The approach used by administration officers was scripted and the text is available elsewhere [22]. The research team then telephoned consenting mothers to provide further details about the study and to arrange for a research assistant to visit mothers in their home or at another location chosen by the mothers. At that visit a written description of the study was provided to mothers, their written informed consent was obtained, and the pre-intervention assessment was completed.

Full details of the research design are provided in the trial protocol [22]. In brief, the trial utilized a pragmatic, preference, randomized, non-inferiority design in which service preferences were elicited from mothers at the time of their recruitment [20,23]. Mothers were informed that the aim of the study was to test whether the new clinic+Internet support program was helpful to mothers and babies. Those who expressed a “strong preference” for clinic+Internet support or for standard home-based support were allocated to their preferred intervention, whereas those without strong preferences were randomly assigned to one of the two conditions. Randomization was based on the service identification number (odd vs even) serially assigned to all infants when they are referred to CaFHS from their birthing hospital (assignment of this number is done by central administrative CaFHS staff who had no involvement in the recruitment of mothers, delivery of the intervention or the analysis of results for the study).

**Figure 1:**
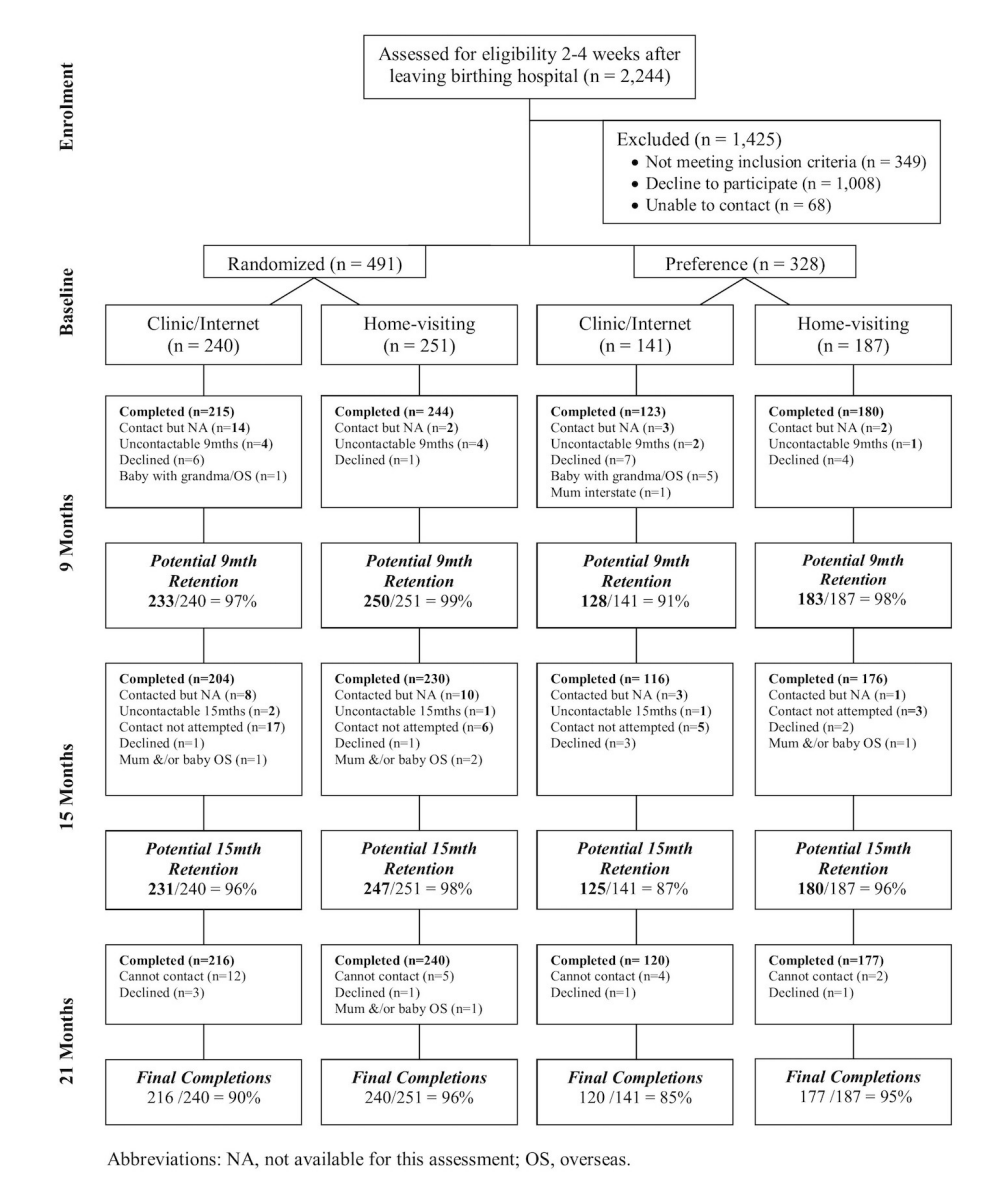
Flow diagram of participants through each stage of the RCT.

The inclusion of the preference trial arm and randomized arms enabled us to examine whether outcomes were not inferior amongst both those who would “choose” a particular mode of service delivery (home-based or clinic-based+Internet) and those willing to be randomized in the study. These lower and higher preference populations may be different and analyzing outcomes from all the groups provide valuable information regarding applicability of the intervention in practice as it indicates more closely what the effects would likely be in real world circumstances [23]. The research team was unable to contact 68 of the 1895 eligible mothers identified. Of the 1827 contacted, 819 agreed to participate (response rate=44.8% (819/1827); see Figure 1). This response rate is consistent with that commonly reported for randomized controlled trials, especially those that are attempting to recruit from almost the entire eligible population, as is the case in this study [24]. Mothers were excluded from participation in the study if (1) they did not have access to the Internet, (2) they required an interpreter, or (3) their nurse or clinician recommended that they not participate due to the presence of problems such as infant ill health, domestic violence or substance abuse [22].

### Clinic+Internet Support

CaFHS aims to complete postnatal health checks within one month of infant births. Mothers in the clinic+Internet group received their postnatal health check at CaFHS community health clinics before being assigned to an online mothers’ group. On average each group was comprised of 12 mothers (range 9-12). Groups were moderated by 1 of 7 qualified CaFHS nurses experienced in facilitating face-to-face mothers’ groups, who had completed a 3-day training program on managing Internet-based mothers’ groups. Groups took place from the time infants were aged approximately 1 month until they were 7 months old [22].

The Internet-based intervention used in the study was developed and implemented by the authors in close collaboration with staff at CaFHS. The aim was to leverage the potential of the Internet to develop a new service model that could provide ongoing professional support to mothers without the need for postnatal home-based help. The website for the intervention was built by independent Web application developers [25] and focus groups were utilized pre-trial to ensure its usability. Intellectual property ownership was based on organizational agreements between the South Australian Department of Health and Ageing and The University of Adelaide and included neither the authors nor the website developers. The affiliations for both organizations were displayed on the login screen.

Mothers could login to the intervention website via computer or mobile device and employed asynchronous text-based communication to exchange information, provide mutual support, and seek help from their nurse facilitator [26]. The “chat” page of the website was designed to be similar to “chat rooms” found on many other websites, including “Facebook,” with the important difference that content was moderated and enhanced by an experienced maternal and child health nurse. This format was utilized instead of the threaded discussion trees common in discussion forum websites because it was more likely to be familiar and hence easier for mothers to use.

Information provided about other mothers in each group was initially limited to first names. Additionally, mothers were told that all members of their group had delivered an infant within a few weeks of each other and all had been enrolled in the group when their infants were approximately one month old. This approach of providing only limited information in the first instance is consistent with the approach used in face-to-face mothers’ groups and addresses issues of privacy and confidentiality. However, also consistent with face-to-face groups, after the groups commenced nurses encouraged mothers to share additional information about themselves on the chat page, which many mothers did.

During the intervention, nurses followed a curriculum that addressed 11 broad topic areas relevant to mothers and infants (eg, sleeping and settling, breastfeeding, and infant development). However, the chronological order in which the topic material was presented to groups in the chat page was flexible and could be varied depending on the nature of the discussions taking place between mothers. Mothers asked questions about a wide range of issues relevant to maternal and infant health including approaches to address maternal tiredness, settling infants, and breast feeding. Mothers also discussed these issues among themselves and offered encouragement to each other when having difficulties in these areas. About 29.9% (114/381) of mothers took up an option to be notified if there was activity on their group’s online chat page. Nurses reviewed all posts twice each week. While doing this they would acknowledge mothers’ comments, encourage discussion between mothers, and provide evidence-based information to support parenting skills. They would also correct misinformation and redirect mothers to relevant online resources both within the intervention website and externally. If necessary, nurses could follow-up with individual participants via email, text messaging, telephone calls, or appointments for face-to-face contact. However, this was rarely necessary.

The online groups functioned in a comparable fashion to face-to-face mothers’ groups that typically took place weekly in community clinics in South Australia. A key issue for both types of mothers’ groups was to ensure that the mothers understood that such interventions are not designed to provide urgent or emergency support. Information about sources of help for the latter was provided on the website and also by nurses during online discussions. Mothers could also access additional parenting information and relevant support telephone numbers (eg, parent helplines) through the intervention website (see Multimedia Appendix 1 for screenshots of the website).

The intervention content did not change during the trial and participants had continual access to the website during the 6 month intervention. There were no scheduled downtimes and the server was monitored in case of outages. Mothers reported any technical difficulties to their nurse moderator or to the research team. Nurses reported problems to the research team. Problems were rare and were resolved either by the research team or by the website developers. A feature allowing mothers and nurses to report problems directly to the website developers was deployed to the website approximately 5 months prior to the end of the trial. The intervention website received some minor updates during the trial such as color changes to website features to enhance visibility, and improved website compatibility with older Web-browsers and mobile devices.

### Home-Based Support

Mothers in the home-based support group received their postnatal health check in their home. This included a health check for mothers and infants, and provision of booklets and pamphlets about maternal care of infants and relevant community-based resources. Home-based support visits by CaFHS nurses were scheduled to last 60-90 minutes.

### Measures

Trained research assistants took written self-assessment questionnaires to mothers in their homes or at another location chosen by the mother. Mothers completed questionnaires when they enrolled in the study, mean age of the child in weeks post randomization being 4.1(SD 1.3) and again when their child was aged 9, 15, and 21 months.

### Maternal Outcomes

Outcomes were chosen according to their priority as outcome goals for the standard CaFHS support program for new mothers in South Australia [22]. The primary outcome was parenting self-competence, which was assessed using two different measurements: the Parenting Stress Index (PSI) Competence subscale (excluding two items assessing parental education, 11 items; range 11-55) [27] and the Karitane Parenting Confidence Scale (15 items; range 0-45) [28,29]. We chose two measures to assess self-competence because different measures focus on somewhat different aspects of the construct of parenting self-competence. Inclusion of two measures made it possible to determine the consistency of the results in the study, regardless of the particular measure used. The analysis of results was undertaken independently for each measure.

The secondary outcomes were maternal social support assessed using PSI Isolation subscale (6 items; range 6-30) [27], the Interpersonal Support Evaluation List – Short Form (ISEL-SF; 16 items; range 0-48) [30], and the Maternal Support Scale, adapted from the Diabetes Support Scale [31] (12 items; range 12-84; Cronbach alpha=.92). Additional secondary analyses examining mother-infant attachment, maternal well-being, and spousal support are reported in Multimedia Appendix 2.

### Child Outcomes

Children’s socioemotional development was assessed at 9, 15, and 21 months using the Ages and Stages Questionnaire–Social-Emotional (ASQ-SE) [32]. The version of the ASQ-SE appropriate for the child’s age was administered at each assessment (9 months: 22 items; range 0-220; 15 and 21 months: 26 items; range 0-380). Children’s verbal development was assessed at 21 months using the MCDI-SF [33]. This measure included a vocabulary checklist that yields a composite score for each checked word (100 items; range 0-100). Minor adaptations were employed with permission from the authors to replace some uncommon American words with equivalent Australian words (eg, candy was changed to lolly).

All questionnaires except for the MCDI-SF [33] utilized Likert-type response options. Higher scores indicate a higher level of problems on the PSI subscales [27] and the ASQ-SE [32], and a lower level of problems on the other questionnaires.

Demographic information included children’s gender, maternal age (in years), education, prenatal employment, housing, and family characteristics (eg, single-parent or two-parent; number of children).

### Ethics Approval

Ethics approval was received from the Women’s and Children’s Health Network Human Research Ethics Committee (REC2368/4/17).

### Missing Data

Although 91.9% (753/819) mothers completed the final assessment, 3.1% (25/819) mothers were missing one or two baseline demographic items, and 36.9% (302/819) mothers were missing one or more outcome measures across the four assessments. To reduce potential bias from missing data, we used multiple imputation by chained equations [34]. We generated 20 data sets that were identical for complete data but could differ for imputed values [35,36]. We used the method of chained equations, randomly sampling the imputed values from the posterior predictive distribution of the missing data [37,38]. Datasets were imputed separately for the clinic+Internet and home-based support groups for randomized and preference participants. Due to non-convergence of imputation models, parental living situation was imputed separately to outcomes and was not utilized in statistical analyses. Subsequent analyses utilized imputed data and adjusted coefficients and variance estimates for variability between datasets according to Rubin’s rules [39].

### Statistical Analysis

All analyses were intent-to-treat. At each assessment point and for each measure, the adjusted mean clinic+Internet group outcome score was compared with the adjusted mean home-based support group outcome score.

The clinic+Internet score on each measure was considered to be “non-inferior” to the home-based support score if the difference between them was less than the score equivalent to 0.25 of the SD of the overall baseline score for the particular measure. This non-inferiority margin is considered a small effect and was established a priori [22,40]. An example of the inferiority margin calculation is as follows. The overall baseline PSI Competence mean (SD) score among all randomized participants was 22.61 (SD 5.24). The non-inferiority margin for this measure was therefore 0.25 x 5.24 (SD 1.31).

We estimated that a sample size of 200 per randomized group would provide 80% power at alpha=.05 to detect a difference of this magnitude [41]. For outcomes in which higher scores indicate more problems, such as the PSI subscales, the clinic+Internet group was considered to be non-inferior to the home-based support group if the upper 95% CI of the difference between the adjusted group means was less than the non-inferiority margin for that measure [42,43]. Outcomes for which higher scores indicate less problems, such as the Karitane Parenting Confidence Scale, non-inferiority was concluded if the lower 95% CI was greater than the relevant non-inferiority margin [42].

The MCDI-SF was completed on a single occasion and outcome data analyzed using multiple linear regression. For all other measures, adjusted mean outcome scores at each assessment were identified using linear generalized estimating equations (GEE) employing exchangeable within-group correlation structures and the Huber-White sandwich estimator [44]. In the models, predictor variables were group (clinic+Internet vs home-based support), time (baseline, 9, 15, 21 months), group by time interaction, and baseline maternal age, highest level of education, employment status and housing, and child’s gender and first child status. Subsequently, a second GEE analysis (with time and group by time interaction variables) was used to identify the size of the difference between adjusted group mean (95% CI) outcome scores at each assessment [42]. All analyses were conducted using Stata 14.1 [34].

Two sensitivity analyses were conducted. The first adjusted for possible clustering according to online group membership by including a dummy variable coding for online-group in the generalized estimating equation used to identify adjusted mean scores (95% CI) for clinic+Internet mothers. The second was a complete case analysis. Results from these sensitivity analyses were very similar to those reported in the manuscript (see Multimedia Appendices 3 and 4).

## Results

The demographic characteristics of mothers in each study group are shown in Table 1. Across the four groups, 47.3% (387/819) of mothers were first-time mothers. However, as previously reported, being a first-time versus experienced mother had little impact on mother’s level of engagement with the intervention [21].

Information about the use of social media, online forums, and parenting websites was collected from all participants at enrolment and again at the 9 month follow-up assessment. At enrolment, on average across the study groups, 38.8% (312/804) of mothers reported frequently (ie, “most days or many times”) using social media to obtain parenting information, 25.7% (210/818) frequently used online forums, and 19.2% (153/795) frequently used parenting websites. At the 9 month follow-up assessment, 42.2% (313/742) of mothers frequently used social media, while only 11.1% (84/760) frequently used online forums and 7.0% (51/733) frequently used parenting websites (see Multimedia Appendix 5 for the frequencies in each study group).

As part of the standard support offered to all mothers in South Australia, during the 6 months following their initial home-based or clinic-based postnatal review, mothers in both study groups could receive additional support from nurses in either or both settings. This additional support could be initiated by nurses or mothers. Table 2 shows that among those randomly assigned to their groups, 1.8% (4/224) of mothers in the clinic+Internet group and 11.4% (28/246) in the home-based support group received 1-2 additional home visits. Also, 37.1% (83/224) of mothers in the clinic+Internet group and 28.5% (70/246) in the home-based support group attended 1-2 additional clinic appointments. In all groups a small percentage of mothers completed more than 4 additional home visits or clinic appointments (Table 2). It is likely that these were mothers or infants who had significant health or psychosocial problems, or had problems in both areas. In general, the patterns of service use were similar for the randomized and preference groups.

The adjusted mean (SE) outcome scores in the clinic+Internet and home-based support groups at each assessment point, the mean (95% CI) difference between these scores, and the non-inferiority cut-off for each measure are shown in Tables 3 and 4. In all groups, all mean outcome scores at baseline were in the “normal range” (Tables 3 and 4).

**Table 1:** Participants’ baseline demographic characteristics.

Characteristic		Randomized		Preference	
		Clinic+Internet	Home-based	Clinic+Internet	Home-based
		(n=240)	(n=251)	(n=141)	(n=187)
First child, n (%)		103 (42.9)	116 (46.2)	63 (44.7)	105 (56.1)
Male child, n (%)		125 (52.1)	126 (50.2)	79 (56.0)	90 (48.1)
Two-parent household, n (%)		232 (96.7)	237 (94.4)	137 (97.2)	183 (97.9)
**Mother’s education^a^, n (%)**					
	University degree	122 (51.0)	123 (49.0)	80 (56.7)	95 (51.0)
	Trade or technical school	65 (27.1)	69 (27.6)	N/A^b^	N/A
	Some or all years of high school	53 (21.9)	59 (23.4)	N/A	N/A
**Mother’s employment, n (%)**					
	Full-time paid employment	136 (56.5)	134 (53.4)	72 (51.1)	108 (57.8)
	Part-time paid employment	72 (30.0)	74 (29.5)	55 (39.0)	60 (32.1)
	Other	32 (13.5)	43 (17.1)	14 (9.9)	19 (10.2)
**Housing, n (%)**					
	Rental	75 (31.3)	83 (33.1)	53 (37.9)	36 (19.3)
	Own home	165 (68.8)	168 (66.9)	88 (62.1)	151 (80.7)
Maternal age, mean (SD)		32.7 (4.7)	32.3 (5.3)	32.5 (5.0)	33.2 (4.5)

^a^Mother’s highest completed education. This was a dichotomous variable (completed university vs no university degree) for preference participants as the three category variable failed to converge during multiple imputation. Complete case distributions of all categories are reported in Multimedia Appendix 4.

^b^N/A: not applicable.

**Table 2:** Proportion of participants receiving additional services during the 6 months following their postnatal review.

Number of services		Randomized		Preference	
		Clinic+Internet	Home-based	Clinic+Internet	Home-based
		(n=224)	(n=246)	(n=121)	(n=177)
**Home, n (%)**					
	0	214 (95.5)	208 (84.6)	114 (94.2)	153 (86.4)
	1-2	4 (1.8)	28 (11.4)	2 (1.7)	17 (9.6)
	3-4	0 (0.0)	4 (1.6)	0 (0.0)	2 (1.1)
	> 4	6 (2.7)	6 (2.4)	5 (4.1)	5 (2.8)
**Clinic, n (%)**					
	0	105 (46.9)	138 (56.1)	62 (51.2)	102 (57.6)
	1-2	83 (37.1)	70 (28.5)	34 (28.1)	40 (22.6)
	3-4	23 (10.3)	25 (10.2)	15 (12.4)	17 (9.6)
	> 4	13 (5.8)	13 (5.3)	10 (8.3)	18 (10.2)

**Table 3:** Randomized participants: adjusted mean (SE), and difference between mean (95% CI) outcome scores. All scores adjusted for child’s gender, number of children, maternal education, maternal employment prior to the birth of her baby, housing situation, and maternal age (years) at baseline.

Outcome assessment				Clinic+Internet (n=240)	Home-based (n=251)		Difference (95% CI)		Non-inferiority criterion^a^	
**Maternal confidence**							
	**Parenting Stress Index – Competence^b^**											
		Baseline		22.64 (0.04)	22.59 (0.04)		0.05 (−0.07 to 0.17)			
		9 months		20.82 (0.04)	20.59 (0.04)		0.23 (0.11 to 0.35)		Upper CI < 1.31	
		15 months		20.22 (0.04)	20.21 (0.04)		0.01 (−0.11 to 0.13)		Upper CI < 1.31	
		21 months		20.33 (0.04)	19.86 (0.04)		0.47 (0.35 to 0.59)		Upper CI < 1.31	
	**Karitane Parenting Confidence Scale**											
		Baseline		40.13 (0.02)	40.08 (0.02)		0.05 (−0.01 to 0.11)			
		9 months		41.79 (0.02)	41.83 (0.02)		−0.04 (−0.10 to 0.02)		Lower CI > −1.05	
		15 months		41.83 (0.02)	41.80 (0.02)		0.02 (−0.04 to 0.08)		Lower CI > −1.05	
		21 months		42.07 (0.02)	42.22 (0.02)		−0.15 (−0.21 to −0.09)		Lower CI > −1.05	
**Maternal social support**												
	**Parenting Stress Index – Isolation^b^**											
		Baseline		11.24 (0.02)	11.13 (0.02)		0.11 (0.05 to 0.17)			
		9 months		11.89 (0.02)	11.49 (0.02)		0.39 (0.33 to 0.45)		Upper CI < 0.85	
		15 months		11.77 (0.02)	11.74 (0.02)		0.03 (−0.04 to 0.09)		Upper CI < 0.85	
		21 months		11.79 (0.02)	11.77 (0.02)		0.01 (−0.05 to 0.08)		Upper CI < 0.85	
	**Interpersonal Support Evaluation List – Short Form**											
		Baseline		40.50 (0.07)	40.74 (0.07)		−0.25 (−0.44 to −0.06)	
		9 months		38.96 (0.07)	39.43 (0.07)		−0.47 (−0.66 to −0.28)		Lower CI > −1.38	
		15 months		38.84 (0.07)	38.64 (0.07)		0.20 (0.01 to 0.39)		Lower CI > −1.38	
		21 months		38.55 (0.07)	39.28 (0.07)		−0.74 (−0.92 to −0.55)		Lower CI > −1.38	
	**Maternal Support Scale**											
		Baseline		74.98 (0.08)	75.85 (0.09)		−0.88 (−1.11 to −0.64)			
		9 months		75.11 (0.08)	76.04 (0.09)		−0.92 (−1.16 to −0.69)		Lower CI > −2.26	
		15 months		75.61 (0.08)	75.56 (0.09)		0.05 (−0.18 to 0.28)		Lower CI > −2.26	
		21 months		75.65 (0.08)	76.30 (0.09)		−0.65 (−0.89 to −0.42)		Lower CI > −2.26	
**Child outcomes**												
	**Ages and Stages Questionnaire – Social-Emotional^b^**											
		9 months		22.09 (0.20)	21.36 (0.22)		0.73 (0.15 to 1.31)		Upper CI < 3.38	
		15 months		24.14 (0.20)	23.74 (0.22)		0.40 (−0.18 to 0.98)		Upper CI < 3.91	
		21 months		23.63 (0.20)	22.36 (0.22)		1.27 (0.69 to 1.85)		Upper CI < 3.98	
	**MacArthur Communication Development Inventories**											
		21 months		29.69 (0.32)	34.45 (0.33)		−4.76 (−5.67 to −3.85)		Lower CI > −5.30^c^	

^a^Non-inferiority is found when the 95% CI of the difference between the means meets the non-inferiority criteria. Non-inferiority is not applicable to baseline scores.

^b^Higher scores indicate more problems.

^c^Failed to achieve non-inferiority.

**Table 4:** Preference participants: adjusted mean (SE), and difference between mean (95% CI) outcome scores. All scores adjusted for child’s gender, number of children, maternal education, maternal employment prior to the birth of her baby, housing situation, and maternal age (years) at baseline.

Outcome assessment			Clinic+Internet (n=141)	Home-based (n=187)	Difference (95% CI)	Non-inferiority criterion^a^
**Maternal confidence**							
	**Parenting Stress Index – Competence^b^**						
		Baseline	22.55 (0.02)	22.83 (0.02)	−0.28 (−0.35 to −0.21)	
		9 months	20.43 (0.02)	20.48 (0.02)	−0.05 (−0.11 to −0.02)	Upper CI < 1.40
		15 months	20.57 (0.02)	20.51 (0.02)	0.06 (−0.01 to 0.13)	Upper CI < 1.40
		21 months	20.31 (0.02)	20.13 (0.02)	0.18 (0.11 to 0.25)	Upper CI < 1.40
	**Parenting Stress Index – Isolation**						
		Baseline	39.97 (0.05)	40.19 (0.03)	−0.21 (−0.32 to −0.10)	
		9 months	41.29 (0.05)	41.92 (0.03)	−0.62 (−0.73 to −0.51)	Lower CI > −1.08
		15 months	41.63 (0.05)	42.14 (0.03)	−0.51 (−0.62 to −0.40)	Lower CI > −1.08
		21 months	41.71 (0.05)	42.35 (0.03)	−0.64 (−0.75 to −0.53)	Lower CI > −1.08
**Maternal social support**							
	**Parenting Stress Index – Isolation^b^**						
		Baseline	11.46 (0.08)	11.12 (0.07)	0.34 (0.14 to 0.54)	
		9 months	12.05 (0.08)	11.21 (0.07)	0.83 (0.64 to 1.03)	Upper CI < 0.96^c^
		15 months	12.33 (0.08)	11.71 (0.07)	0.62 (0.42 to 0.82)	Upper CI < 0.96
		21 months	12.20 (0.08)	11.56 (0.07)	0.65 (0.45 to 0.85)	Upper CI < 0.96
	**Interpersonal Support Evaluation List – Short Form”**						
		Baseline	40.41 (0.10)	40.93 (0.09)	−0.52 (−0.78 to −0.25)	
		9 months	38.77 (0.10)	39.53 (0.09)	−0.76 (−1.02 to −0.49)	Lower CI > −1.58
		15 months	38.29 (0.10)	39.22 (0.09)	−0.94 (−1.20 to −0.67)	Lower CI > −1.58
		21 months	38.38 (0.10)	39.23 (0.09)	−0.85 (−1.11 to −0.58)	Lower CI > −1.58
	**Maternal Support Scale**						
		Baseline	73.93 (0.13)	77.09 (0.11)	−3.16 (−3.49 to −2.82)	
		9 months	74.74 (0.13)	75.72 (0.11)	−0.99 (−1.32 to −0.65)	Lower CI > −2.42
		15 months	74.37 (0.13)	76.30 (0.11)	−1.94 (−2.27 to −1.60)	Lower CI > −2.42
		21 months	75.11 (0.13)	76.69 (0.11)	−1.58 (−1.92 to −1.25)	Lower CI > −2.42
**Child outcomes**							
	**Ages and Stages Questionnaire – Social-Emotional^b^**							
		9 months	22.25 (0.37)	21.42 (0.27)	0.83 (−0.07 to 1.72)	Upper CI < 3.34
		15 months	23.69 (0.37)	22.00 (0.27)	1.69 (0.80-2.58)	Upper CI < 3.87
		21 months	24.72 (0.37)	22.08 (0.27)	2.64 (1.75-3.54)	Upper CI < 4.21
	**MacArthur Communication Development Inventories**							
		21 months	32.38 (0.74)	33.33 (0.62)	−0.95 (−2.84 to 0.94)	Lower CI > −5.61

^a^Non-inferiority is found when the 95% CI of the difference between the means meets the non-inferiority criteria. Non-inferiority is not applicable to baseline scores.

^b^Higher scores indicate more problems.

^c^Failed to achieve non-inferiority.

As noted earlier, for each measure we compared the size of the difference between the mean clinic+Internet and home-based support group scores with the a priori non-inferiority cut-off score identified for the particular measure. For example, Table 3 shows the difference between the PSI Competence scores at 9 months was 0.23 (95% CI=0.11-0.35). Since the upper CI for this difference is less than this measure’s non-inferiority cut-off score of 1.31, the outcome for the clinic+Internet group was considered to be non-inferior to that of the home-based support group at this assessment.

For randomly assigned participants, at each follow-up assessment, all maternal outcome scores and all ASQ-SE scores in the clinic+Internet group were non-inferior to the corresponding scores in the home-based support group (Table 3). However, the difference between the MCDI-SF adjusted mean scores was −4.79 (95% CI=−5.66 to −3.92) with the lower 95% CI extending beyond the non-inferiority cut-off of −5.30.

Results for mothers in the preference groups were similar to those for randomly assigned mothers with two exceptions (Table 4). First, in the preference groups the adjusted mean MCDI-SF score in the clinic+Internet group was not inferior to the score in the home-based support group. Secondly, the adjusted mean PSI Isolation score in the clinic+Internet group, for which higher scores indicate more problems, was inferior to the home-based support group mean score at 9 months (adjusted mean difference = 0.80; upper 95% CI=0.99, exceeding the non-inferiority cut-off of 0.96). However, the clinic+Internet group score on this measure was not inferior to the home-based support score at 15 and 21 months (Table 4).

## Discussion

### Principal Findings

When infants were aged 9, 15, and 21 months, outcomes for mothers and infants who received a clinic-based health check+Internet support were generally not inferior to those who received home-based support. The primary outcome of parenting self-competence was not inferior on scores for both the PSI Competence scale and the Karitane Parenting Confidence Scale. There were two exceptions to the general pattern of non-inferiority. First, children of mothers randomly assigned to receive clinic+Internet support, but not those assigned on the basis of their preference, had inferior MCDI scores assessing their verbal development at 21 months. However, the difference was not inferior for randomized and preference groups in the complete case analyses (see Multimedia Appendix 4). Second, for mothers assigned to the clinic+Internet group on the basis of their preference, but not those randomly assigned, PSI Isolation scores assessing maternal social isolation were inferior at 9 months. At all other time points, non-inferiority was met for all outcomes in both randomized and preference groups.

The results suggest that clinic-based support combined with a nurse-moderated Internet-based group intervention delivered when infants are aged 1-7 months can achieve comparable outcomes for mothers and infants to those achieved by universal home-based support. This is important because Internet delivery has the potential to allow nurses to provide ongoing support services without the need to travel to mothers’ homes, reduce costs of ‘no-show’ visits, and allow one nurse to work with more families during a single day. For mothers, Internet delivery enables access to reliable, evidence-based “just-in-time” information and nursing support without the need to attend fixed-time appointments in clinics that may be geographically distant from their homes. With regards to engagement in the present study, randomized mothers logged into the website a median of 9 times (IQR=1–25) during the first 6 weeks of the intervention, and a median of 10 times (IQR=0–39) during the remaining 19 weeks. The median time to mothers’ last login was 4.9 months [21].

Qualitative feedback provided by mothers at follow-up assessments showed that the intervention components most valued by mothers were access to helpful advice and information when needed, contact with their nurse moderator, and support from other new mothers with similar experiences. It was notable that mothers accepted and valued the regular presence of an experienced nurse in the forum. This was evident in mothers’ comments when advice was offered from one mother to another and they reflected on whether their nurse would agree with the advice being offered, much as occurs in discussion between mothers in face-to-face groups moderated by nurses. Importantly, it did not appear that the presence of a nurse moderator inhibited mothers in their discussions with each other but rather provided reassurance that advice being shared was consistent with what would be suggested by a professional in the area. In this context, ‘misinformation’ tended to be one of emphasizing one approach over another or a recommended style of handling a problem rather than being grossly incorrect advice.

The clinic+Internet program has the potential to improve and maintain population reach, help ensure quality control of the information provided, and facilitate access to support services during an infant’s first year. In contrast to home-based support with its focus on helping individual mothers in their homes, group-based online support programs also have the potential to provide ready access for new mothers to nurse-moderated peer support.

Delivering nurse support to mothers and infants via the Internet has three other potential benefits. First, it provides the opportunity for nurses to readily track mothers’ level of engagement with different components of a support program. Second, by using simple quizzes and other feedback from mothers, nurses can gauge mothers’ ongoing knowledge acquisition. This could enable nurses to more accurately target services to mothers and infants most in need of support. Third, the provision of easily accessible information about developmental milestones and early childhood health problems has the potential to facilitate mothers’ early recognition of their child’s developmental or health problems. In the past, developmental screening was undertaken in health services or community clinics at fixed intervals according to the age of infants and children. However, current evidence in child health and development emphasizes the importance of replacing fixed age reviews with a more continuous approach sensitive to developmental variability of children, and combining this with increased awareness of developmental issues at a population level [45]. This poses an enormous challenge for current methods of universal service provision.

### Strengths and Limitations

One of the main strengths of the study was its pragmatic nature. The entire RCT was conducted within a routine service setting using existing administrative infrastructure to allocate participants and deliver the intervention as part of normal service delivery. Other strengths include the “preference” design which showed the results were similar for mothers randomly assigned to their study groups and those assigned on the basis of their preference. This similarity increases the likelihood that results would apply generally to mothers utilizing a similar nurse-moderated Internet-based group intervention during the post-natal period [23]. Potential limitations include evidence that participating mothers were from a somewhat more socially advantaged group. However, the Australian Bureau of Statistics shows that 42.0% of Australian women aged 25-34 have university qualifications compared with 51.4% (421/819) of participants in this study, suggesting that participating mothers may not be greatly more advantaged than the general female population [46]. Finally, a cost-effective analysis lies outside the scope of this manuscript but will be reported in the future.

### Conclusion

Results from the study suggest that clinic-based support combined with a nurse-moderated Internet-based group intervention delivered to new mothers can achieve comparable outcomes to those achieved by universal home-based support. Improving early childhood outcomes has been recognized as a policy priority internationally [47,48]. Achieving this requires cost-effective interventions with wide population reach, which can enhance early childhood health and well-being at a population level. In many countries, including Australia, population-level maternal and infant services are provided via relatively expensive home-based nurse support programs. However, it is possible that for many carers, population-wide services could be just as effectively provided by clinic-based nurses supported by Internet-based programs, especially among lower risk groups who may comprise up to 70% of the population [49]. This could assist the better targeting of home-based programs toward carers and infants who need this more intensive level of support.

## Appendix

Multimedia Appendix 1 Screenshots of the intervention website.

Multimedia Appendix 2 Analyses of additional secondary outcomes (mother-infant attachment, maternal well-being, and spousal support).

Multimedia Appendix 3 Analyses of maternal confidence and social support, and of the child outcomes, adjusting for possible online-group clustering in the clinic+Internet group.

Multimedia Appendix 4 Complete case analyses of maternal confidence and social support, and of the child outcomes.

Multimedia Appendix 5 Mothers’ use of online resources for parenting information.

Multimedia Appendix 6 CONSORT-eHEALTH V1.6.

## Abbreviations

ASQ-SE: Ages and Stages Questionnaire–Social-Emotional
CaFHS: Child and Family Health Service
MCDI: MacArthur Communicative Development Inventories
N/A: not applicable
PSI: Parenting Stress Index
ISEL-SF: Interpersonal Support Evaluation List–Short Form
