# Supplementary material for: Nurse-Moderated Internet-Based Support for New Mothers: Non-Inferiority, Randomized Controlled Trial
Source: J Med Internet Res. 2017 Jul 24;19(7):e258. doi: 10.2196/jmir.6839 (PMC5547246; doi:10.2196/jmir.6839)
Supplement: Multimedia Appendix 1 [file jmir_v19i7e258_app1.pdf]

Multimedia Appendix 1: Screenshots of the intervention website.

Note. Participants and conversations depicted are for demonstration purposes only; they were not actual participants.

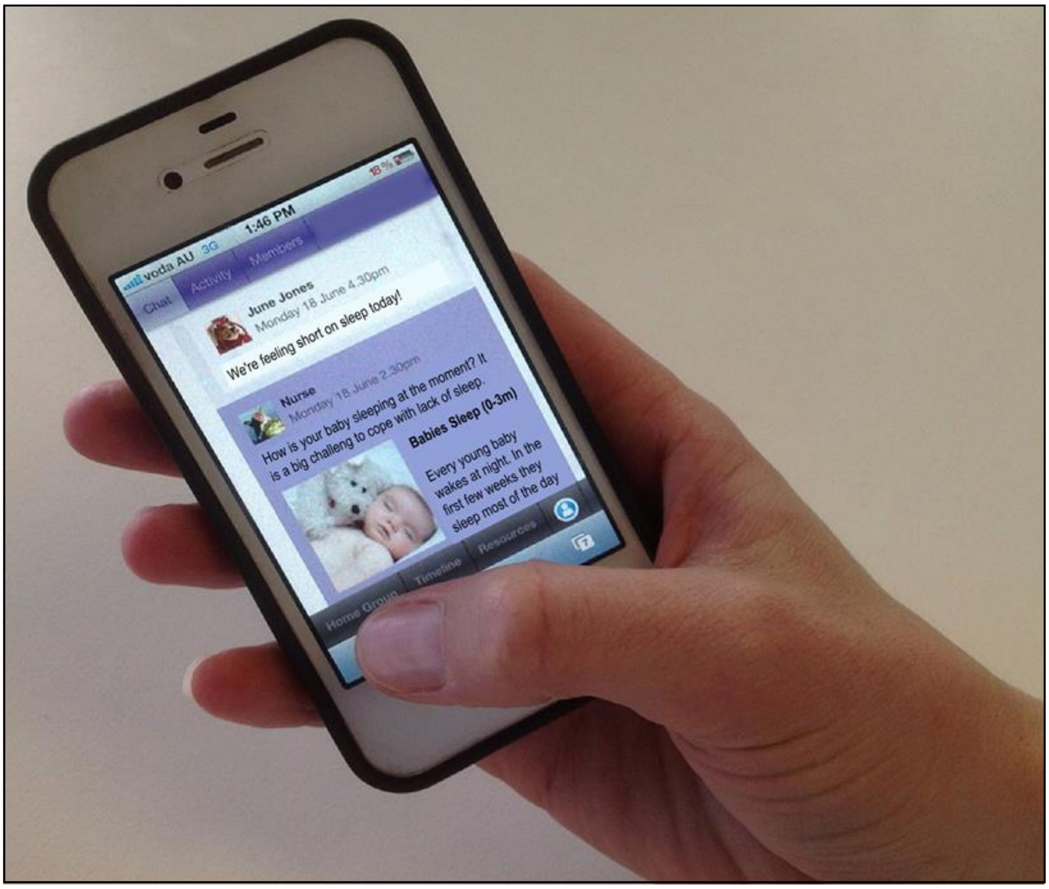

Figure 1. The intervention website could be accessed via desktop computers and mobile devices.

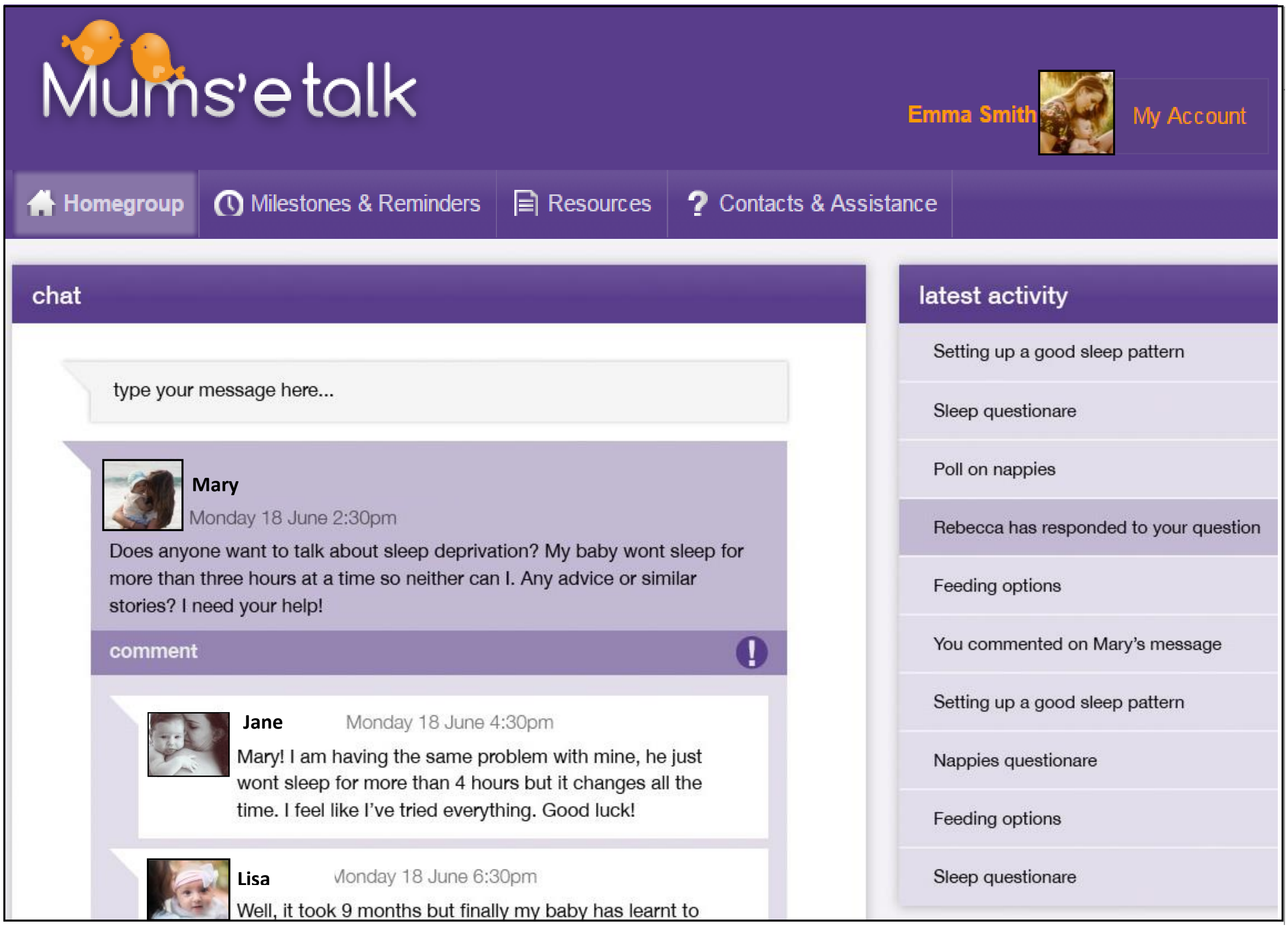

Figure 2. The "Chat" section of the intervention website provided mothers and nurses with access to the online mothers' group moderated by a CaFHS nurse. The format of this section was similar to 'chat rooms' on other websites, such as 'Facebook', with comments appearing in text boxes below the original post. All group members could see all posts and comments.

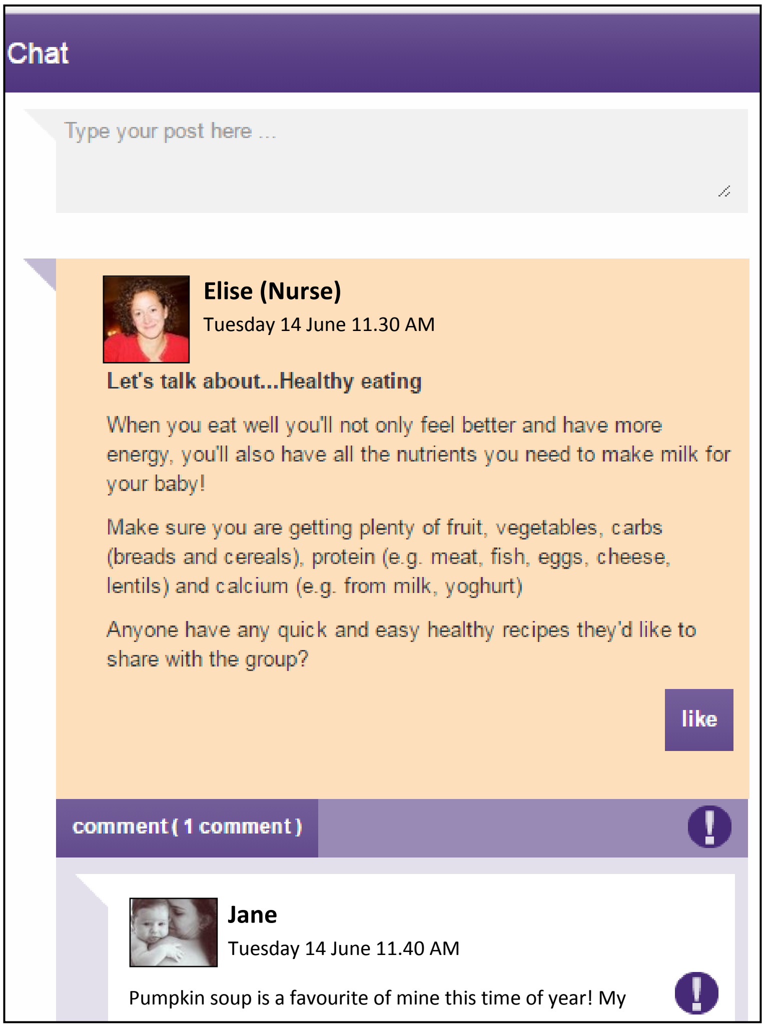

Figure 3. In addition to ‘chatting’ online with mothers, nurse moderators utilised the Chat page to post information topics relevant to mothers and infants. Mothers could discuss these topics by commenting on the post.

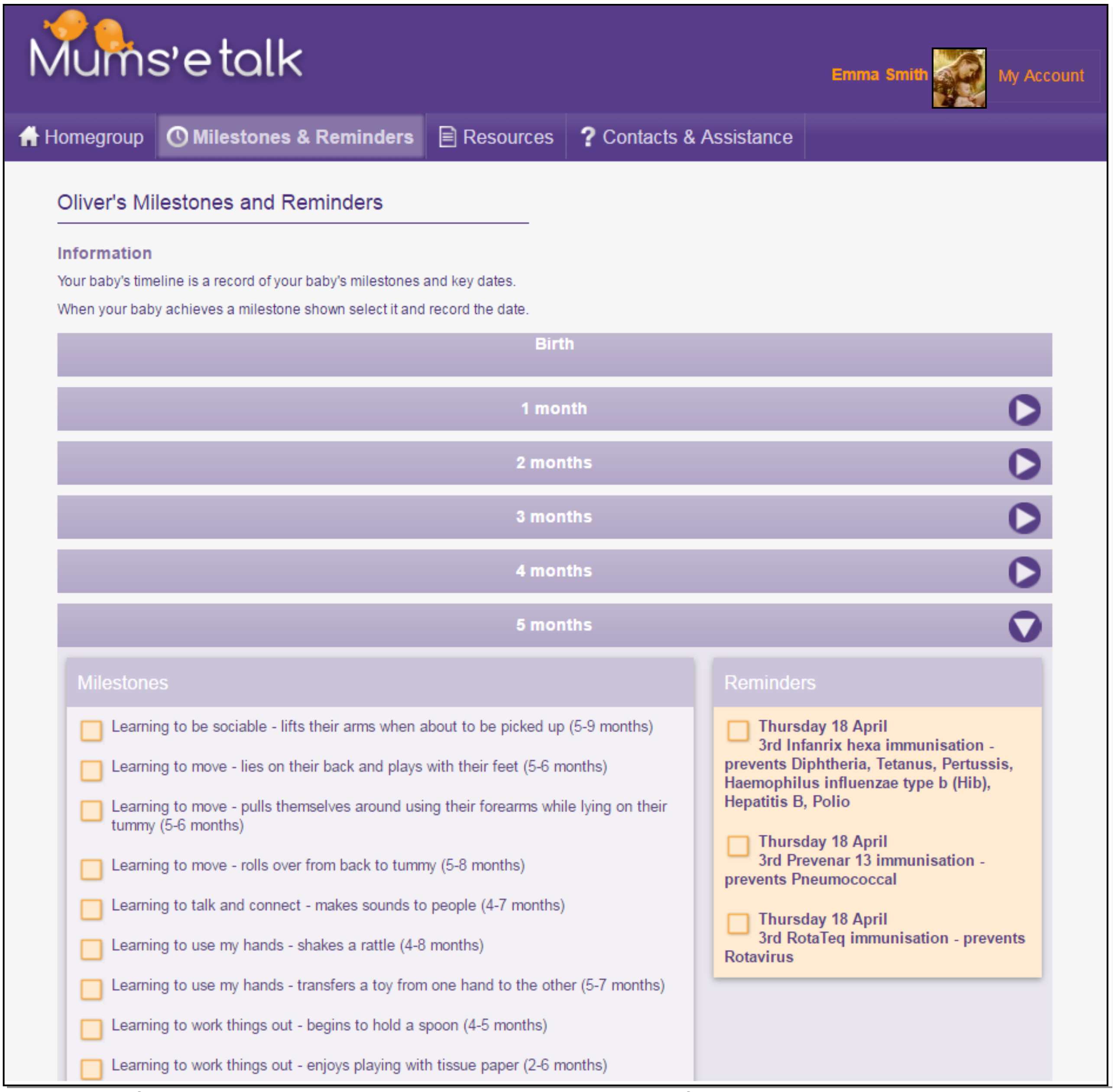

Figure 4. Information about developmental milestones and infant health reminders were provided in a separate tab in the website.

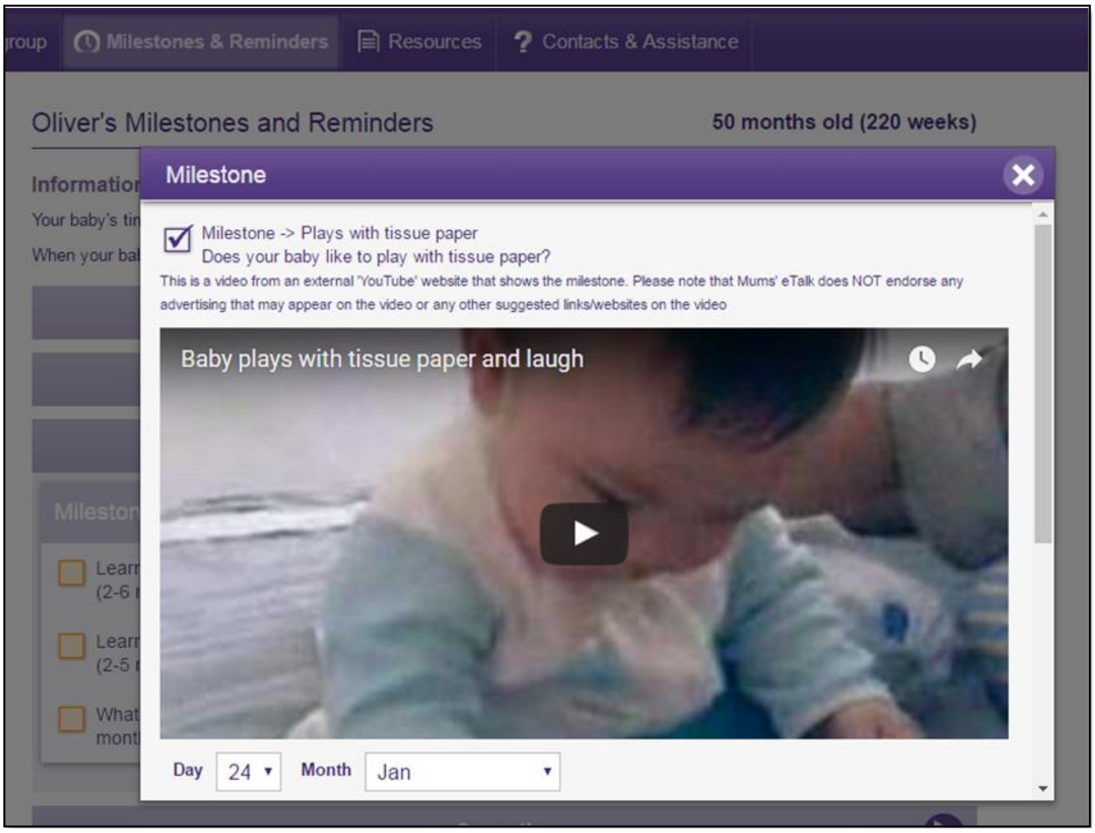

Figure 5. Mothers could view examples of baby milestones and record their baby's achieved milestones and reminders in the Milestones & Reminders section of the website.

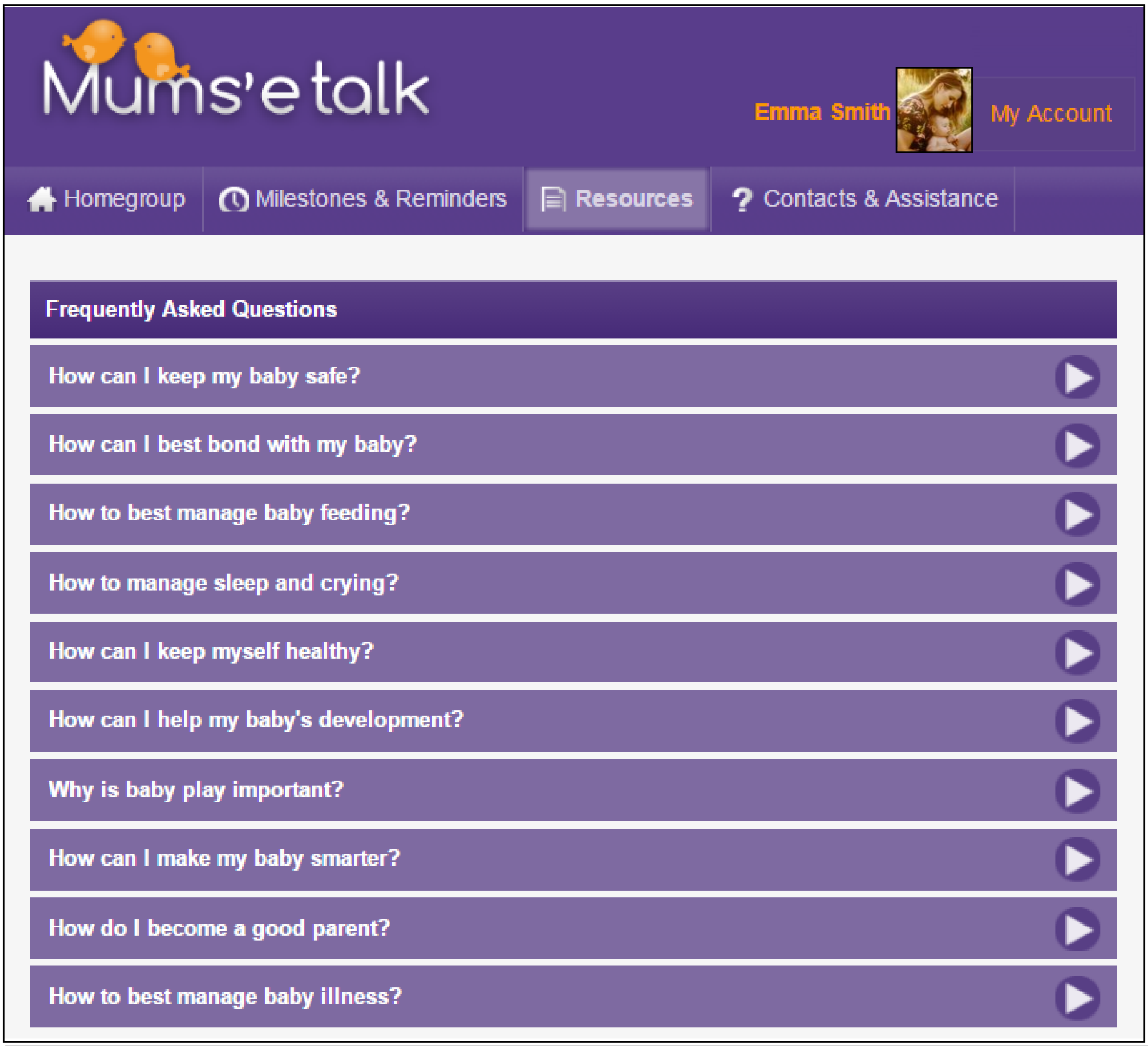

Figure 6. Mothers could access additional parenting information in the form of Frequently Asked Questions. Content was developed by the authors and staff from the South Australian Child and Family Health Service.

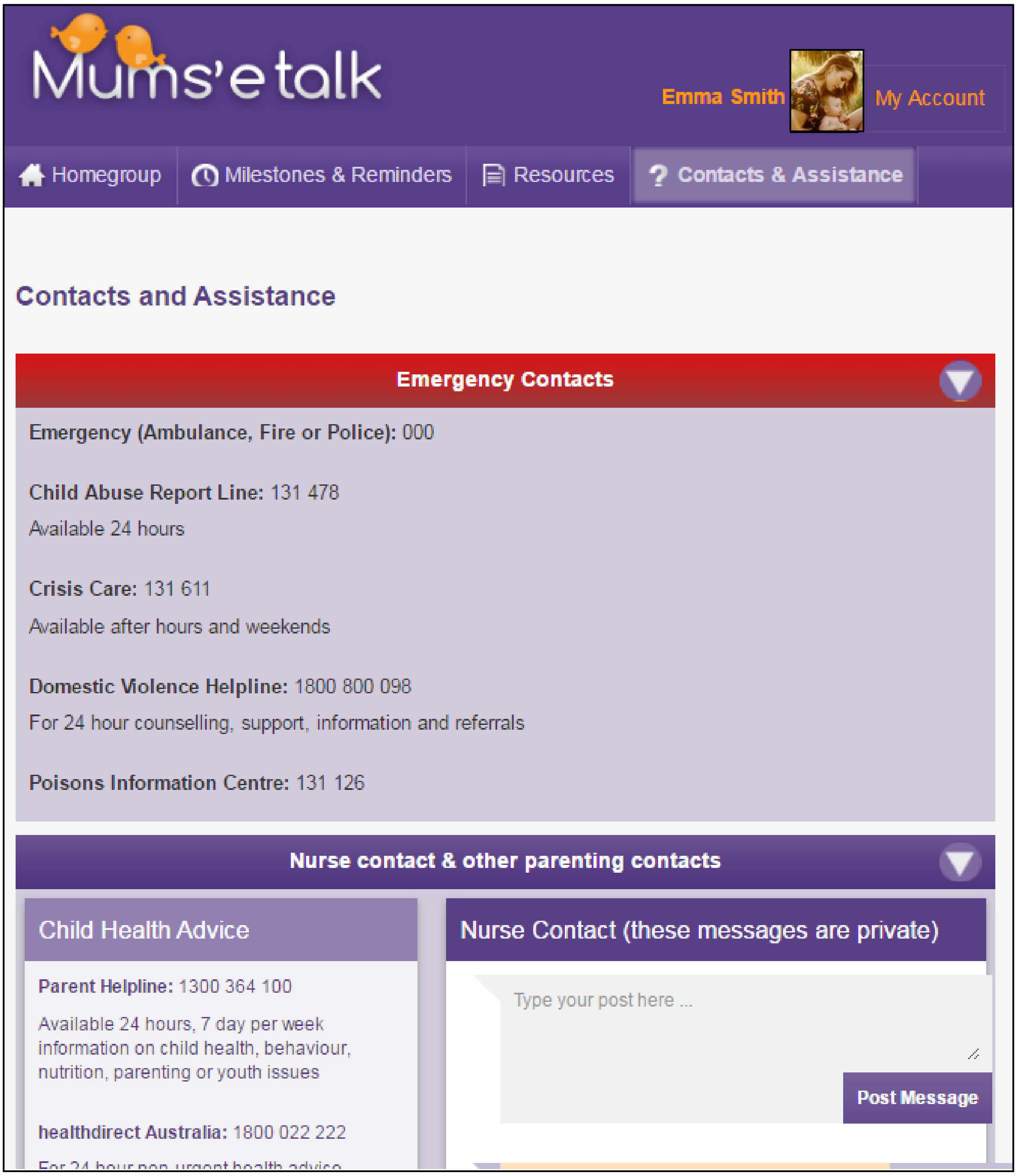

Figure 7. Mothers were provided with a list of helpful phone numbers and could privately message their nurse facilitator if necessary.

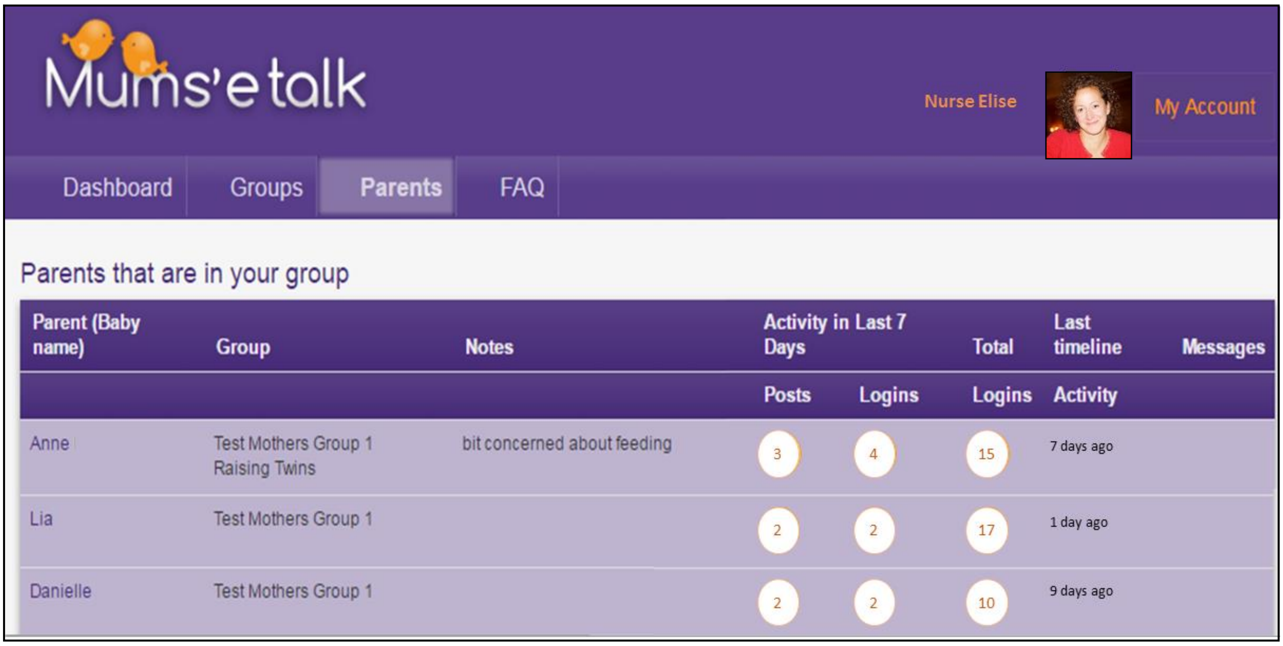

Figure 8. The nurses' view of the intervention website contained summaries of mothers' activities and additional tools to facilitate moderation of the mothers' groups.
