# Supplementary material for: Nurse-Moderated Internet-Based Support for New Mothers: Non-Inferiority, Randomized Controlled Trial
Source: J Med Internet Res. 2017 Jul 24;19(7):e258. doi: 10.2196/jmir.6839 (PMC5547246; doi:10.2196/jmir.6839)
Supplement: Multimedia Appendix 2 [file jmir_v19i7e258_app2.pdf]

Multimedia Appendix 2: Analyses of additional secondary outcomes (mother-infant attachment, maternal well-being, and spousal support).

Table 1. Participants' baseline demographic characteristics (n [%])

**Characteristic**	**Randomised**		**Preference**	
	**Clinic+ Internet (n = 240)**	**Home-based (n = 251)**	**Clinic+ Internet (n = 141)**	**Home-based (n = 187)**
First child (%)	116 (42.8)	103 (46.2)	63 (44.7)	105 (56.1)
Male child (%)	125 (52.1)	126 (50.2)	79 (56.0)	90 (48.1)
Two-parent household (%)	232 (96.7)	237 (94.4)	137 (97.2)	183 (97.9)
Mother's completed education[a] (%)				
University	122 (50.6)	123 (49.1)	80 (57.0)	96 (51.3)
Trade/Technical school	66 (27.3)	70 (27.7)	NA	NA
Some/all years of high school	53 (22.0)	58 (23.2)	NA	NA
Mother's employment (%)				
Full-time paid employment	136 (56.6)	134 (53.4)	72 (51.1)	108 (57.8)
Part-time paid employment	72 (30.1)	74 (29.5)	55 (39.0)	60 (32.1)
Other	32 (13.4)	43 (17.1)	14 (9.9)	19 (10.2)
Housing (%)				
Rental	75 (31.3)	83 (33.1)	54 (38.0)	36 (19.3)
Own home	165 (68.8)	168 (66.9)	87 (62.0)	151 (80.7)
Maternal age (mean [SD])	32.69 (4.68)	32.24 (5.18)	32.50 (5.04)	33.22 (4.47)

Abbreviation: SD, standard deviation.

[a] Mother's highest completed education. This was a dichotomous variable (completed university vs no university degree) for preference participants as the imputation model failed to converge with the three category variable. Complete case distributions are reported in Appendix C.

Table 2. Adjusted mean (SE), and difference between mean (95% CI) outcome scores[a] for randomised participants (clinic+Internet group n = 240, home-based group n = 251)

Outcome Assessment	Clinic+ Internet	Home-based	Mean Difference (95% CI)	Non-Inferiority Criterion[b]
**Mother-Infant Attachment**				
PSI Attachment[c]				
Baseline	10.23 (0.03)	10.15 (0.04)	0.08 (-0.02 to 0.17)	
9 months	10.03 (0.03)	9.56 (0.04)	0.47 (0.37 to 0.57)	Upper CI < 0.86
15 months	9.78 (0.03)	9.92 (0.04)	-0.14 (-0.24 to -0.04)	Upper CI < 0.86
21 months	10.41 (0.03)	10.10 (0.04)	0.31 (0.22 to 0.41)	Upper CI < 0.86
Maternal Attachment[d]				
Baseline	85.53 (0.11)	84.79 (0.11)	0.74 (0.44 to 1.05)	
9 months	85.24 (0.11)	85.09 (0.11)	0.15 (-0.16 to 0.45)	Lower CI > -1.73
15 months	84.18 (0.11)	85.16 (0.11)	0.01 (-0.29 to 0.32)	Lower CI > -1.73
21 months	83.47 (0.11)	83.59 (0.11)	-0.12 (-0.43 to 0.19)	Lower CI > -1.73
**Maternal Wellbeing**				
Everyday Feelings[c]				
Baseline	10.56 (0.04)	10.76 (0.04)	-0.20 (-0.31 to -0.10)	
9 months	10.99 (0.04)	10.83 (0.04)	0.15 (0.05 to 0.26)	Upper CI < 1.21
15 months	10.98 (0.04)	11.05 (0.04)	-0.07 (-0.18 to 0.03)	Upper CI < 1.21
21 months	10.86 (0.04)	11.17 (0.04)	-0.31 (-0.41 to -0.20)	Upper CI < 1.21
PSI Role Restriction[c]				
Baseline	16.67 (0.05)	17.09 (0.05)	-0.42 (-0.56 to -0.28)	
9 months	17.16 (0.05)	16.84 (0.05)	0.31 (0.17 to 0.45)	Upper CI < 1.20
15 months	16.86 (0.05)	16.93 (0.05)	-0.07 (-0.21 to 0.07)	Upper CI < 1.20
21 months	17.10 (0.05)	16.75 (0.05)	0.35 (0.21 to 0.49)	Upper CI < 1.20
**Maternal Spousal Support**				
PSI Spouse[ce]				
Baseline	13.37 (0.04)	14.04 (0.04)	-0.67 (-0.77 to -0.57)	
9 months	14.92 (0.04)	14.81 (0.04)	0.11 (0.01 to 0.21)	Upper CI < 1.04
15 months	14.84 (0.04)	15.00 (0.04)	-0.16 (-0.26 to -0.05)	Upper CI < 1.04
21 months	15.00 (0.04)	15.00 (0.04)	-0.00 (-0.10 to 0.10)	Upper CI < 1.04

Abbreviations: CI, confidence interval; Everyday Feelings, Everyday Feelings Questionnaire; PSI, Parenting Stress Index; SE, standard error.

[a] All scores adjusted for child’s gender, number of children, maternal education, maternal employment prior to the birth of her baby, housing situation, and maternal age (years) at baseline.

[b] Non-inferiority is found when the 95% Confidence Interval of the difference between the means meets the non-inferiority criteria. Non-inferiority is not applicable to baseline scores.

[c] Higher scores indicate more problems.

[d] Maternal Postnatal Attachment Scale.

[e] Parenting Stress Index Spouse subscale scores were not imputed when missing for single mothers.

Table 3. Adjusted mean (SE), and difference between mean (95% CI) outcome scores[a] for preference participants (clinic+Internet group n = 141, home-based group n = 187)

Outcome Assessment	Clinic+ Internet	Home-based	Mean Difference (95% CI)	Non-Inferiority Criterion[b]
**Mother-Infant Attachment**				
PSI Attachment[c]				
Baseline	10.44 (0.04)	10.41 (0.04)	0.03 (-0.09 to 0.15)	
9 months	9.82 (0.04)	9.74 (0.04)	0.08 (-0.04 to 0.19)	Upper CI < 0.82
15 months	10.12 (0.04)	9.96 (0.04)	0.17 (0.05 to 0.29)	Upper CI < 0.82
21 months	10.09 (0.04)	9.99 (0.04)	0.11 (-0.01 to 0.23)	Upper CI < 0.82
Maternal Attachment[d]				
Baseline	85.18 (0.09)	84.39 (0.08)	0.78 (0.54 to 1.03)	
9 months	85.29 (0.09)	84.62 (0.08)	0.67 (0.43 to 0.91)	Lower CI > -1.69
15 months	83.93 (0.09)	83.32 (0.08)	0.61 (0.37 to 0.86)	Lower CI > -1.69
21 months	83.54 (0.09)	83.15 (0.08)	0.39 (0.15 to 0.64)	Lower CI > -1.69
**Maternal Wellbeing**				
Everyday Feelings[c]				
Baseline	10.32 (0.08)	10.40 (0.07)	-0.08 (-0.30 to 0.14)	
9 months	10.81 (0.08)	10.48 (0.07)	0.33 (0.11 to 0.55)	Upper CI < 1.26
15 months	10.64 (0.08)	10.35 (0.07)	0.28 (0.06 to 0.50)	Upper CI < 1.26
21 months	10.96 (0.08)	10.50 (0.07)	0.46 (0.24 to 0.68)	Upper CI < 1.26
PSI Role Restriction[c]				
Baseline	17.32 (0.10)	17.45 (0.09)	-0.13 (-0.38 to 0.13)	
9 months	17.06 (0.10)	16.94 (0.09)	0.12 (-0.14 to 0.37)	Upper CI < 1.17
15 months	17.18 (0.10)	17.10 (0.09)	0.08 (-0.18 to 0.34)	Upper CI < 1.17
21 months	16.57 (0.10)	17.08 (0.09)	-0.51 (-0.77 to -0.26)	Upper CI < 1.17
**Maternal Spousal Support**				
PSI Spouse[ce]				
Baseline	13.94 (0.09)	13.47 (0.07)	0.47 (0.24 to 0.70)	
9 months	14.81 (0.09)	14.79 (0.07)	0.01 (-0.22 to 0.24)	Upper CI < 1.06
15 months	14.98 (0.09)	14.83 (0.07)	0.15 (-0.08 to 0.38)	Upper CI < 1.06
21 months	15.14 (0.09)	14.84 (0.07)	0.29 (0.06 to 0.52)	Upper CI < 1.06

Abbreviations: CI, confidence interval; Everyday Feelings, Everyday Feelings Questionnaire; PSI, Parenting Stress Index; SE, standard error.

[a] All scores adjusted for child's gender, number of children, maternal education, maternal employment prior to the birth of her baby, housing situation, and maternal age (years) at baseline.

[b] Non-inferiority is found when the 95% Confidence Interval of the difference between the means meets the non-inferiority criteria. Non-inferiority is not applicable to baseline scores.

[c] Higher scores indicate more problems.

[d] Maternal Postnatal Attachment Scale.

[e] Parenting Stress Index Spouse subscale scores were not imputed when missing for single mothers.
