# Supplementary material for: Nurse-Moderated Internet-Based Support for New Mothers: Non-Inferiority, Randomized Controlled Trial
Source: J Med Internet Res. 2017 Jul 24;19(7):e258. doi: 10.2196/jmir.6839 (PMC5547246; doi:10.2196/jmir.6839)
Supplement: Multimedia Appendix 3 [file jmir_v19i7e258_app3.pdf]

Multimedia Appendix 3: Analyses of maternal confidence and social support, and of the child outcomes, adjusting for possible online-group clustering in the clinic+internet group.

Table 1. Adjusted mean (SE), and difference between mean (95% CI) outcome scores[a] for randomised participants (clinic+internet group n = 240, home-based group n = 251)

Outcome Assessment	Clinic+ Internet	Home-based	Mean Difference (95% CI)	Non-Inferiority Criterion[b]
**Maternal Confidence**				
PSI Competence[c]				
Baseline	22.64 (0.12)	22.59 (0.06)	0.05 (-0.21 to 0.31)	
9 months	20.82 (0.12)	20.59 (0.06)	0.23 (-0.04 to 0.49)	Upper CI < 1.31
15 months	20.22 (0.12)	20.21 (0.06)	0.01 (-0.25 to 0.27)	Upper CI < 1.31
21 months	20.33 (0.12)	19.86 (0.06)	0.47 (0.21 to 0.73)	Upper CI < 1.31
Karitane Parent Confidence				
Baseline	40.13 (0.07)	40.08 (0.02)	0.05 (-0.10 to 0.19)	
9 months	41.79 (0.07)	41.83 (0.02)	-0.04 (-0.18 to 0.11)	Lower CI > -1.05
15 months	41.83 (0.07)	41.80 (0.02)	0.02 (-0.12 to 0.17)	Lower CI > -1.05
21 months	42.07 (0.07)	42.22 (0.02)	-0.15 (-0.30 to -0.01)	Lower CI > -1.05
**Maternal Social Support**				
PSI Isolation[c]				
Baseline	11.24 (0.06)	11.13 (0.03)	0.11 (-0.03 to 0.24)	
9 months	11.89 (0.06)	11.49 (0.03)	0.39 (0.26 to 0.53)	Upper CI < 0.85
15 months	11.77 (0.06)	11.74 (0.03)	0.03 (-0.11 to 0.16)	Upper CI < 0.85
21 months	11.79 (0.06)	11.77 (0.03)	0.01 (-0.12 to 0.15)	Upper CI < 0.85
ISEL-SF				
Baseline	40.50 (0.13)	40.74 (0.08)	-0.25 (-0.55 to 0.05)	
9 months	38.96 (0.13)	39.43 (0.08)	-0.47 (-0.77 to -0.17)	Lower CI > -1.38
15 months	38.84 (0.13)	38.64 (0.08)	0.20 (-0.10 to 0.50)	Lower CI > -1.38
21 months	38.55 (0.13)	39.28 (0.08)	-0.74 (-1.03 to -0.44)	Lower CI > -1.38
Maternal Support Scale				
Baseline	74.98 (0.17)	75.85 (0.12)	-0.88 (-1.28 to -0.47)	
9 months	75.11 (0.17)	76.04 (0.12)	-0.92 (-1.33 to -0.52)	Lower CI > -2.26
15 months	75.61 (0.17)	75.56 (0.12)	0.05 (-0.35 to 0.46)	Lower CI > -2.26
21 months	75.65 (0.17)	76.30 (0.12)	-0.65 (-1.06 to -0.25)	Lower CI > -2.26
**Child Outcomes**				
Ages & Stages-SE[c]				
9 months	22.09 (0.34)	21.36 (0.28)	0.73 (-0.13 to 1.59)	Upper CI < 3.38
15 months	24.14 (0.34)	23.74 (0.28)	0.40 (-0.46 to 1.27)	Upper CI < 3.91
21 months	23.63 (0.34)	22.36 (0.28)	1.27 (0.41 to 2.14)	Upper CI < 3.98
Communication (MCDI)				
21 months	29.69 (0.70)	34.45 (0.44)	-4.76 (-6.39 to -3.14)	Lower CI > -5.30[d]

Abbreviations: Ages & Stages-SE, Ages and Stages Questionnaire – Social-Emotional; CI, confidence interval; ISEL-SF, Interpersonal Support Evaluation List – Short Form; Karitane Parent Confidence, Karitane Parenting Confidence Scale; MCDI, MacArthur Communication Development Inventories; PSI, Parenting Stress Index; SE, standard error.

[a] Adjusted for baseline demographic characteristics as described in the manuscript. Intervention participants' scores also adjusted for intervention group (categorical).

[b] Non-inferiority is found when the 95% Confidence Interval of the difference between the means meets the non-inferiority criteria. Non-inferiority is not applicable to baseline scores.

[c] Higher scores indicate more problems.

[d] Failed to achieve non-inferiority.

Table 2. Adjusted mean (SE), and difference between mean (95% CI) outcome scores[a] for preference participants (clinic+internet group n = 141, home-based group n = 187)

Outcome Assessment	Clinic+ Internet	Home-based	Mean Difference (95% CI)	Non-Inferiority Criterion[b]
**Maternal Confidence**				
PSI Competence[c]				
Baseline	22.55 (0.19)	22.83 (0.03)	-0.28 (-0.65 to 0.09)	
9 months	20.43 (0.19)	20.48 (0.03)	-0.05 (-0.42 to 0.33)	Upper CI < 1.40
15 months	20.57 (0.19)	20.51 (0.03)	0.06 (-0.31 to 0.43)	Upper CI < 1.40
21 months	20.31 (0.19)	20.13 (0.03)	0.18 (-0.19 to 0.55)	Upper CI < 1.40
Karitane Parent Confidence				
Baseline	39.97 (0.13)	40.19 (0.03)	-0.21 (-0.48 to 0.05)	
9 months	41.29 (0.13)	41.92 (0.03)	-0.62 (-0.89 to -0.36)	Lower CI > -1.08
15 months	41.63 (0.13)	42.14 (0.03)	-0.51 (-0.78 to -0.25)	Lower CI > -1.08
21 months	41.71 (0.13)	42.35 (0.03)	-0.64 (-0.90 to -0.38)	Lower CI > -1.08
**Maternal Social Support**				
PSI Isolation[c]				
Baseline	11.46 (0.15)	11.12 (0.07)	0.34 (0.01 to 0.67)	
9 months	12.05 (0.15)	11.21 (0.07)	0.83 (0.51 to 1.16)	Upper CI < 0.96[d]
15 months	12.33 (0.15)	11.71 (0.07)	0.62 (0.29 to 0.95)	Upper CI < 0.96
21 months	12.20 (0.15)	11.56 (0.07)	0.65 (0.32 to 0.98)	Upper CI < 0.96[d]
ISEL-SF				
Baseline	40.41 (0.28)	40.93 (0.09)	-0.52 (-1.10 to 0.06)	
9 months	38.77 (0.28)	39.53 (0.09)	-0.76 (-1.33 to -0.18)	Lower CI > -1.58
15 months	38.29 (0.28)	39.22 (0.09)	-0.94 (-1.51 to -0.36)	Lower CI > -1.58
21 months	38.38 (0.28)	39.23 (0.09)	-0.85 (-1.42 to -0.27)	Lower CI > -1.58
Maternal Support Scale				
Baseline	73.93 (0.36)	77.09 (0.11)	-3.16 (-3.90 to -2.41)	
9 months	74.74 (0.36)	75.72 (0.11)	-0.99 (-1.73 to -0.24)	Lower CI > -2.42
15 months	74.37 (0.36)	76.30 (0.11)	-1.94 (-2.68 to -1.19)	Lower CI > -2.42[d]
21 months	75.11 (0.36)	76.69 (0.11)	-1.58 (-2.33 to -0.84)	Lower CI > -2.42
**Child Outcomes**				
Ages & Stages-SE[c]				
9 months	22.25 (0.69)	21.42 (0.25)	0.83 (-0.60 to 2.26)	Upper CI < 3.34
15 months	23.69 (0.69)	22.00 (0.25)	1.69 (0.26 to 3.12)	Upper CI < 3.87
21 months	24.72 (0.69)	22.08 (0.25)	2.64 (1.21 to 4.07)	Upper CI < 4.21
Communication (MCDI)				
21 months	32.38 (1.04)	33.33 (0.75)	-0.95 (-3.47 to 1.56)	Lower CI > -5.61

Abbreviations: Ages & Stages-SE, Ages and Stages Questionnaire – Social-Emotional; CI, confidence interval; ISEL-SF, Interpersonal Support Evaluation List – Short Form; MCDI, MacArthur Communication Development Inventories; PSI, Parenting Stress Index; SE, standard error.

[a] Adjusted for baseline demographic characteristics as described in the manuscript. Intervention participants' scores also adjusted for intervention group (categorical).

[b] Non-inferiority is found when the 95% Confidence Interval of the difference between the means meets the non-inferiority criteria. Non-inferiority is not applicable to baseline scores.

[c] Higher scores indicate more problems.

[d] Failed to achieve non-inferiority.
