# Supplementary material for: Nurse-Moderated Internet-Based Support for New Mothers: Non-Inferiority, Randomized Controlled Trial
Source: J Med Internet Res. 2017 Jul 24;19(7):e258. doi: 10.2196/jmir.6839 (PMC5547246; doi:10.2196/jmir.6839)
Supplement: Multimedia Appendix 4 [file jmir_v19i7e258_app4.pdf]

Multimedia Appendix 4: Complete case analyses of maternal confidence and social support, and of the child outcomes.

Table 1. Number of mothers with complete data at the baseline assessment (n, [%])

**Study Group**	**Participants with Complete Baseline Data**
Randomised arm	
Clinic + internet (n=240)	231 (96.3)
Home-based (n=251)	249 (99.2)
Preference	
Clinic + internet (n=141)	139 (98.6)
Home-based (n=187)	180 (96.3)

Table 2. Mothers' baseline demographic characteristics (n [%], mean[SD])

**Characteristic**	**Randomised**		**Preference**	
	**Clinic+ Internet (n = 231)**	**Home-based (n = 249)**	**Clinic+ Internet (n = 136)**	**Home-based (n = 180)**
First child (%)	98 (42.2)	116 (46.6)	63 (46.3)	102 (56.7)
Male child (%)	120 (51.9)	126 (50.6)	76 (55.9)	90 (50.0)
Two-parent household (%)	223 (97.0)	231 (94.3)	132 (97.1)	175 (97.8)
Mother's highest completed education (%)				
Completed university	117 (50.6)	122 (49.0)	78 (57.4)	92 (51.1)
Technical school/some university	64 (27.7)	69 (27.7)	40 (29.4)	59 (32.8)
Some/all years of high school	50 (21.6)	58 (23.3)	18 (13.2)	29 (16.1)
Mother's employment (%)				
Full-time paid employment	131 (56.7)	132 (53.0)	71 (52.2)	104 (57.8)
Part-time paid employment	71 (30.7)	74 (29.7)	52 (38.2)	58 (32.2)
Other	29 (12.6)	43 (17.3)	13 (9.6)	18 (10.0)
Housing (%)				
Rental	72 (31.2)	83 (33.3)	51 (37.5)	36 (20.0)
Own home	159 (68.8)	166 (66.7)	85 (62.5)	144 (80.0)
Maternal age (mean[SD])	31.82 (4.58)	31.43 (5.07)	31.64 (4.90)	32.29 (4.34)

Abbreviation: SD, standard deviation.

Table 3. Adjusted mean (SE), and difference between mean (95% CI) outcome scores[a] for randomised participants (clinic+internet group n = 240, home-based group n = 251)

Outcome Assessment	Clinic+ Internet	Home-based	Difference (95% CI)[b]	Non-Inferiority Criterion[c]
**Maternal Confidence**				
PSI Competence[d] (n=480)				
Baseline	22.65 (0.05)	22.59 (0.05)	0.06 (-0.07 to 0.19)	
9 months	20.60 (0.05)	20.61 (0.05)	-0.01 (-0.15 to 0.12)	Upper CI < 1.31
15 months	20.01 (0.05)	20.20 (0.05)	-0.19 (-0.33 to -0.06)	Upper CI < 1.31
21 months	20.05 (0.05)	19.79 (0.05)	0.26 (0.12 to 0.39)	Upper CI < 1.31
Karitane Parent Confidence (n=480)				
Baseline	40.23 (0.02)	40.08 (0.02)	0.15 (0.08 to 0.22)	
9 months	41.98 (0.03)	41.82 (0.02)	0.15 (0.08 to 0.22)	Lower CI > -1.06
15 months	42.13 (0.03)	41.87 (0.02)	0.25 (0.18 to 0.33)	Lower CI > -1.06
21 months	42.29 (0.03)	42.23 (0.02)	0.07 (0.00 to 0.14)	Lower CI > -1.06
**Maternal Social Support**				
PSI Isolation[d] (n=480)				
Baseline	11.22 (0.02)	11.13 (0.02)	0.09 (0.04 to 0.15)	
9 months	11.79 (0.02)	11.52 (0.02)	0.28 (0.22 to 0.34)	Upper CI < 0.86
15 months	11.54 (0.02)	11.72 (0.02)	-0.18 (-0.24 to -0.12)	Upper CI < 0.86
21 months	11.58 (0.02)	11.76 (0.02)	-0.18 (-0.24 to -0.12)	Upper CI < 0.86
ISEL-SF (n=480)				
Baseline	40.54 (0.07)	40.75 (0.07)	-0.21 (-0.39 to -0.03)	
9 months	39.15 (0.07)	39.44 (0.07)	-0.29 (-0.48 to -0.10)	Lower CI > -1.38
15 months	39.43 (0.07)	38.78 (0.07)	0.64 (0.45 to 0.84)	Lower CI > -1.38
21 months	39.00 (0.07)	39.33 (0.07)	-0.32 (-0.51 to -0.13)	Lower CI > -1.38
Maternal Support Scale (n=480)				
Baseline	75.14 (0.07)	75.85 (0.07)	-0.71 (-0.91 to -0.51)	
9 months	75.49 (0.07)	76.07 (0.07)	-0.58 (-0.79 to -0.38)	Lower CI > -2.25
15 months	76.45 (0.07)	75.93 (0.07)	0.53 (0.32 to 0.73)	Lower CI > -2.25
21 months	76.38 (0.07)	76.47 (0.07)	-0.09 (-0.30 to 0.12)	Lower CI > -2.25
**Child Outcomes**				
Ages & Stages-SE[d] (n=464)				
9 months	21.70 (0.23)	21.32 (0.23)	0.38 (-0.26 to 1.02)	Upper CI < 3.30
15 months	23.86 (0.23)	23.62 (0.24)	0.24 (-0.41 to 0.89)	Upper CI < 3.82
21 months	23.08 (0.23)	22.44 (0.23)	0.64 (0.00 to 1.28)	Upper CI < 3.98
Communication (MCDI)[e] (n=445)				
21 months	30.22 (0.33)	34.24 (0.33)	-4.02 (-4.95 to -3.09)	Lower CI > -5.33

Abbreviations: Ages & Stages-SE, Ages and Stages Questionnaire – Social-Emotional; CI, confidence interval; ISEL-SF, Interpersonal Support Evaluation List – Short Form; Karitane Parent Confidence, Karitane Parenting Confidence Scale; MCDI, MacArthur Communication Development Inventories; PSI, Parenting Stress Index; SE, standard error.

[a] Participants had complete baseline demographic data and at least one outcome assessment (this is the minimum requirement for an individual to be included in the GEE analyses). All scores adjusted for baseline demographic characteristics as described in the manuscript.

[b] Difference calculated using t-tests.

[c] Non-inferiority is found when the indicated upper or lower 95% Confidence Interval of the difference between the means meets the non-inferiority criteria. Non-inferiority is not applicable to baseline scores.

[d] Higher scores indicate more problems.

[e] The MCDI was completed on a single occasion and data analysed using multiple regression analysis.

Table 4. Adjusted mean (SE), and difference between mean (95% CI) outcome scores[a] for preference participants (clinic+internet group n = 141; home-based group n = 187)

Outcome Assessment	Clinic+ Internet	Home-based	Difference (95% CI)[b]	Non-Inferiority Criterion[c]
**Maternal Confidence**				
PSI Competence[d] (n=316)				
Baseline	22.48 (0.03)	22.82 (0.03)	-0.35 (-0.43 to -0.26)	
9 months	20.12 (0.03)	20.41 (0.03)	-0.29 (-0.37 to -0.20)	Upper CI < 1.41
15 months	20.20 (0.03)	20.40 (0.03)	-0.19 (-0.28 to -0.11)	Upper CI < 1.41
21 months	19.96 (0.03)	20.12 (0.03)	-0.15 (-0.24 to -0.06)	Upper CI < 1.41
Karitane Parent Confidence (n=315)				
Baseline	40.03 (0.04)	40.23 (0.03)	-0.20 (-0.31 to -0.10)	
9 months	41.80 (0.04)	42.04 (0.03)	-0.24 (-0.35 to -0.13)	Lower CI > -1.07
15 months	41.98 (0.04)	42.23 (0.03)	-0.25 (-0.36 to -0.14)	Lower CI > -1.07
21 months	42.10 (0.05)	42.38 (0.03)	-0.28 (-0.39 to -0.17)	Lower CI > -1.07
**Maternal Social Support**				
PSI Isolation[d] (n=316)				
Baseline	11.31 (0.07)	11.09 (0.07)	0.23 (0.03 to 0.42)	
9 months	11.65 (0.08)	11.02 (0.07)	0.63 (0.43 to 0.83)	Upper CI < 0.97
15 months	11.98 (0.08)	11.51 (0.06)	0.47 (0.27 to 0.67)	Upper CI < 0.97
21 months	11.86 (0.08)	11.42 (0.07)	0.43 (0.23 to 0.64)	Upper CI < 0.97
ISEL-SF (n=314)				
Baseline	40.55 (0.11)	40.95 (0.10)	-0.41 (-0.69 to -0.12)	
9 months	39.52 (0.12)	39.82 (0.09)	-0.29 (-0.58 to -0.01)	Lower CI > -1.59
15 months	38.98 (0.12)	39.48 (0.09)	-0.50 (-0.80 to -0.21)	Lower CI > -1.59
21 months	38.93 (0.12)	39.38 (0.09)	-0.45 (-0.75 to -0.15)	Lower CI > -1.59
Maternal Support Scale (n=316)				
Baseline	73.98 (0.13)	77.14 (0.12)	-3.17 (-3.53 to -2.81)	
9 months	75.65 (0.15)	76.13 (0.12)	-0.48 (-0.85 to -0.10)	Lower CI > -2.47
15 months	75.46 (0.15)	76.65 (0.12)	-1.20 (-1.57 to -0.82)	Lower CI > -2.47
21 months	76.04 (0.15)	76.92 (0.13)	-0.88 (-1.26 to -0.49)	Lower CI > -2.47
**Child Outcomes**				
Ages & Stages-SE[d] (n=299)				
9 months	20.71 (0.35)	21.09 (0.25)	-0.38 (-1.21 to 0.44)	Upper CI < 3.27
15 months	21.28 (0.36)	21.59 (0.25)	-0.31 (-1.15 to 0.54)	Upper CI < 3.61
21 months	22.44 (0.36)	22.20 (0.26)	0.24 (-0.62 to 1.09)	Upper CI < 4.16
Communication (MCDI)[e] (n=288)				
21 months	33.89 (0.88)	33.14 (0.70)	0.75 (-1.44 to 2.94)	Lower CI > -5.60

Abbreviations: Ages & Stages-SE, Ages and Stages Questionnaire – Social-Emotional; CI, confidence interval; ISEL-SF, Interpersonal Support Evaluation List – Short Form; Karitane Parent Confidence, Karitane Parenting Confidence Scale; MCDI, MacArthur Communication Development Inventories; PSI, Parenting Stress Index; SE, standard error.

[a] Participants had complete baseline demographic data and at least one outcome assessment (this is the minimum requirement for an individual to be included in the GEE analyses). All scores adjusted for baseline demographic characteristics as described in the manuscript.

[b] Difference between clinic+internet and home-based group adjusted means was calculated using t-tests.

[c] Non-inferiority is found when the indicated upper or lower 95% Confidence Interval of the difference between the means meets the non-inferiority criteria. Non-inferiority is not applicable to baseline scores.

[d] Higher scores indicate more problems.

[e] The MCDI was completed on a single occasion and data analysed using multiple regression analysis.
