# Supplementary material for: Nurse-Moderated Internet-Based Support for New Mothers: Non-Inferiority, Randomized Controlled Trial
Source: J Med Internet Res. 2017 Jul 24;19(7):e258. doi: 10.2196/jmir.6839 (PMC5547246; doi:10.2196/jmir.6839)
Supplement: Multimedia Appendix 5 [file jmir_v19i7e258_app5.pdf]

Multimedia Appendix 5: Mothers' use of online resources for parenting information.

Table. Proportion [n (%)][a] of mothers using online resources for parenting information.

**Online Source**	**Randomised**		**Preference**	
	**Clinic+internet (n = 240)**	**Home-based (n = 251)**	**Clinic+internet (n = 141)**	**Home-based (n = 187)**
Social media				
Baseline assessment	97 (41.1)	92 (37.1)	64 (47.1)	59 (32.1)
9 month follow-up	100 (41.2)	85 (35.6)	57 (47.9)	71 (41.3)
Online forums				
Baseline assessment	73 (30.4)	64 (25.5)	38 (27.0)	35 (18.8)
9 month follow-up	24 (11.2)	28 (11.5)	13 (10.7)	19 (10.6)
Parenting websites				
Baseline assessment	46 (19.7)	43 (17.3)	34 (25.4)	30 (16.8)
9 month follow-up	19 (9.2)	13 (5.5)	7 (6.0)	12 (6.9)

[a] There are a small number of missing responses in some cells.
